# Unique Anatomical Features of the Discoid Lateral Meniscus via Three-Dimensional MRI

**DOI:** 10.7759/cureus.46188

**Published:** 2023-09-29

**Authors:** Akinori Nekomoto, Masakazu Ishikawa, Shunya Tsuji, Yasuteru Shimamura, Naoyuki Kitamura, Goki Kamei, Kyohei Nakata, Naofumi Hashiguchi, Atsuo Nakamae, Nobuo Adachi

**Affiliations:** 1 Department of Orthopaedic Surgery, Hiroshima University Hospital, Hiroshima, JPN; 2 Department of Orthopaedic Surgery, Faculty of Medicine, Kagawa University, Takamatsu, JPN; 3 Department of Radiology, Kasumi Clinic, Hiroshima, JPN; 4 Department of Orthopaedic Surgery, Graduate School of Biomedical and Health Sciences, Hiroshima University, Hiroshima, JPN

**Keywords:** the ratio of the medial to lateral anterior-to-posterior length (l/m ratio), anatomical feature, 3-dimensional image, magnetic resonance imaging, discoid lateral meniscus

## Abstract

Background

The discoid lateral meniscus (DLM) is a unique anatomical variant characterized by a larger, thicker lateral meniscus. For clinical diagnosis of DLM, coronal and sagittal slices in two-dimensional (2D) MRI and arthroscopic imaging are typically employed. However, evaluating the entire shape of the DLM is challenging due to the limited views and details provided by these methods. Three-dimensional (3D) visualization with MRI offers a more comprehensive view of the entire meniscus. The purpose of this study was to demonstrate the entire shape of a DLM using 3D images and unveil its unique characteristics.

Methods

The study population consisted of 31 knees diagnosed with DLM through arthroscopic examination at our hospital between 2017 and 2021. This group comprised 20 males (65%) and 11 females (35%), with ages ranging from 9 to 49 years (mean age, 24.2 years). Furthermore, a control group of 43 knees without DLM was included for comparative analysis. This control group consisted of 22 males (51%) and 21 females (49%), with ages ranging from 9 to 69 years (mean age, 28.5 years). 3D images of the medial meniscus (MM) and lateral meniscus (LM) were reconstructed from 1.5T-MRI images with semi-automatic segmentation using free software. From the coordinate information, the anterior-to-posterior lengths of the MM and LM were obtained, and the medial-to-lateral anterior-to-posterior length (L/M ratio) ratio was calculated and compared with the value of the non-DLM population.

Results

Our method allows for the detailed delineation of the DLM's unique morphology. The DLM group exhibited a significantly smaller L/M ratio compared to the non-DLM group (DLM: 0.66±0.06, non-DLM: 0.74±0.05, p<0.001).

Conclusions

Reconstructed 3D images could help to demonstrate the whole morphology of DLM and reveal its unique features, in which DLM shows a significantly smaller L/M ratio as compared to non-DLM.

## Introduction

The discoid lateral meniscus (DLM) represents a distinctive anatomical variation characterized by a larger and thicker lateral meniscus (LM) [1]. Incidence varies from 0.4% to 16.6%, being a common disorder in Eastern Asian populations [2]. Watanabe M et al. classified its morphology as incomplete, complete, or Wrisberg ligament types [3]. MRI serves as a fundamental tool for diagnosing DLMs in clinical practice, with multiple quantitative criteria having been proposed for this purpose. Silverman JM et al. [4] suggested that the presence of DLM can be determined if three or more 5-mm-thick, contiguous sagittal images show continuity of the meniscus between the anterior and posterior horns. Araki Y et al. [5] stated that DLM can be diagnosed if the transverse width at the mid-portion of the meniscal body exceeds 14 mm, regardless of tibial width. Samoto N et al. [6] presented two useful parameters for diagnosing a discoid meniscus: (1) the ratio of the meniscus to the tibia (RMT), which is the ratio of the minimum meniscal width to the maximum tibial width on the coronal slice, and (2) the percent coverage of the meniscus (PCM), the ratio of the sum of the widths of the anterior and posterior horns to the meniscal diameter on the sagittal slice displaying the maximum meniscal diameter. The most accurate diagnostic thresholds were identified as RMT ≥ 20% or PCM ≥ 75%. However, applying these criteria can pose challenges in cases with concomitant meniscal tears and displacement, as seen in radial, longitudinal, and complex tears [7]. Regarding treatment, arthroscopic partial meniscectomy, with or without repair, is commonplace; however, postoperative outcomes frequently fall short of expectations [8, 9]. Intriguingly, treated DLMs may have poorer prognoses compared to normally shaped menisci [10], underscoring the challenges in treatment. Therefore, to execute appropriate surgical intervention and to secure successful clinical outcomes, a detailed evaluation of the precise anatomy and pathology of the DLM is crucial for each patient. While two-dimensional (2D) MRI images often fall short of capturing complete meniscal morphology, three-dimensional (3D) MRI has emerged to comprehensively quantify meniscal volume and positioning. Notably, no study has explored DLM morphology using 3D MRI or evaluated its unique anatomical features. We posit that leveraging patient-specific 3D MRI represents a pivotal advance toward accurate anatomical assessment of DLM. This study employs MRI to reconstruct patient-specific 3D DLM and non-DLM images via free software segmentation. The aim is to assess DLM 3D morphology and pinpoint its unique anatomical features.

## Materials and methods

Participants

This study received approval from the Ethical Committee for Clinical Research of Hiroshima University (IRB ID# E-1228), and informed consent was acquired from all participants included in the study. The study population comprised 31 consecutive knees diagnosed with DLM and 43 consecutive knees without DLM, serving as a control population; these diagnoses were confirmed by arthroscopic surgeries performed at our hospital between 2017 and 2021. DLM was identified as RMT ≥ 0.20 in the coronal MRI image, as outlined by Samoto N et al., and further verified by arthroscopy. Additionally, cases identified as DLM during arthroscopy were included in the DLM group, even if an RMT ≥ 0.20 was not observed on MRI. Furthermore, the DLM group was divided into subgroups according to the presence or absence of tears and the presence of complete or incomplete DLM. Complete DLM was defined as RMT > 0.32 as the cutoff value reported by Choi SH et al. [11]. The DLM group consisted of 20 males and 11 females aged 9-49 years (mean age, 24.2 years). A group of age-matched control patients without DLM who underwent anterior cruciate ligament reconstruction or other surgeries (e.g., meniscectomy) at the same time were included as a non-DLM group in this study. The non-DLM group comprised 22 males and 21 females aged 9-69 years (mean age, 28.5 years). Patients with previous meniscal surgery or those who could not be divided by software were excluded from the study population.

MRI scanning protocol

MRI examinations were performed using a 1.5-T MRI scanner with a dedicated knee coil (Achieva 1.5T; Philips Medical Systems, Netherlands) at a joint research facility. The knee was positioned at 15° flexion and 0° rotation. These knees were assessed using coronal T2-weighted spin-echo images (repetition time, 4243 ms; echo time, 70 ms), sagittal proton density-weighted spin-echo images (PDWI) (2137/10), and axial PDWI (2389/12). The section thickness was 3 mm for the axial, coronal, and sagittal views. In addition, 3D MRI scanning by PDWI (1500/28) was performed with 0.7 mm slice thickness and 0.35 mm interslice gap. Only the axial plane was scanned using this technique, and the total scanning time was 3 min 12 sec. The 3D reconstruction technology was used to obtain images at any level in the coronal and sagittal planes.

MRI data segmentation, processing, and analysis

**Figure 1:**
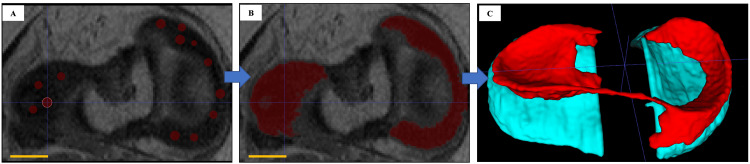
The 3D menisci and cartilage images were generated using ITK-SNAP. A: Multiple arbitrary points were plotted within the range of the meniscus. The bar represents 10 mm. B: The software automatically delineated the meniscus. The bar represents 10 mm. C: Pressing the "update" button in the 3D display panel generated a 3D view. 3D: Three dimensional; ITK-SNAP: Insight Segmentation and Registration Toolkit - Segmentation and Normalization Analysis Program.

MR images were pseudonymized and converted from digital imaging and communications in medicine (DICOM) to the Neuroimaging Informatics Technology Initiative (NIFTI). The cartilage of the tibia plateau and menisci were then semi-automatically segmented using an active contour evolution algorithm implemented in the ITK-SNAP software (ITK-SNAP 3.8.0, http://itksnap.org). 3D surface images of the medial and lateral menisci were reconstructed using the software, as described in a previous report [12]. The images of the menisci were segmented using the thresholding function with manual editing performed by a trained orthopaedic surgeon. The 3D menisci and cartilage images were then generated and optimized by limited smoothing (Figure 1).

**Figure 2:**
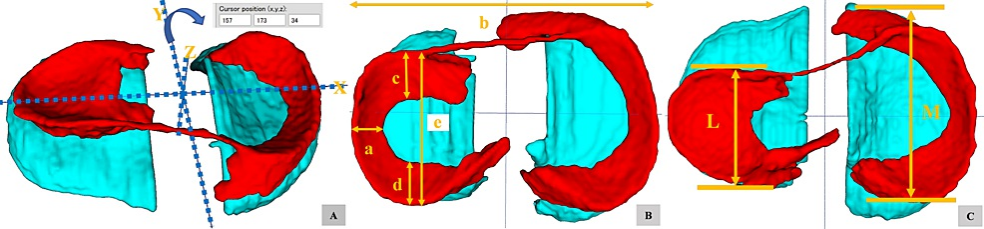
Each parameter is measured using the 3D image on ITK-SNAP as the coordinate system. A: The position is displayed in X-Y-Z coordinates in a 3D image. B: The RMT and PCM are measured according to previous research, with measurements taken when the 3D image is viewed from the top (middle). RMT = a/b, PCM = (c+d)/e C: The L/M ratio is calculated by dividing the anterior to posterior diameter of the lateral meniscus by that of the medial meniscus. L/M ratio = L/M RMT: Ratio of the Meniscus to the Tibia; PCM: Percent Coverage of the Meniscus; 3D: Three Dimensional; ITK-SNAP: Insight Segmentation and Registration Toolkit - Segmentation and Normalization Analysis Program.

Concerning the indices of meniscus size, two quantitative parameters reported by Samoto N et al. [6] were measured using a 3D image in ITK-SNAP, utilizing a coordinate system (Figure 2A). These parameters are (1) RMT, which is the ratio of the minimum meniscal width to the maximum tibial width in the 3D image, and (2) PCM, the ratio of the sum of the widths of the anterior and posterior horns to the meniscal diameter in a sagittal 3D image (Figure 2B). Based on the observation of the entire 3D image of the menisci, we introduced a novel index: (3) the ratio between the anterior-to-posterior diameters of the lateral and medial meniscus (L/M ratio) (Figure 2C). Two orthopedic surgeons, Atsuo Nakamae and Masakazu Ishikawa, performed the measurements in a randomized order, twice by each rater, with at least a 30-day interval between measurements. Observers selected the image displaying the articular surface of the tibial plateau viewed from above and calculated the three parameters in planar coordinates using ITK-SNAP software.

Statistical analysis

Patient demographic data were compared between the two groups using Pearson's χ2 test with Yate's continuity correction, and three parameters (RMT, PCM, and L/M ratio) were compared between the two groups using the Mann-Whitney U test. These parameters were also statistically analyzed for subgroups with and without tears and subgroups with complete or incomplete DLM. The Mann-Whitney U test was applied for two subgroup comparisons. Intra- and inter-observer reliabilities were analyzed using intraclass correlation coefficients (ICCs). Statistical significance was defined as a 95% CI for hazard ratios not including 1.0, and the alpha was set to 0.05.

## Results

Demographic data of the non-DLM and DLM groups are shown in Table 1. No significant differences in age and sex were observed between the groups. According to a DLM report, there were 10 cases of complete DLM and 21 cases of incomplete DLM in the previous group [11]. Furthermore, nine cases of DLM with tears were included in the DLM group (Table 1). Two of the nine cases with tears had complete DLM, while the remaining cases had incomplete DLM.

**Table 1:** Patient’s demographic data. DLM: Discoid lateral meniscus.

	non-DLM group (n=43)	DLM group (n=31)	P-value
Age (years)	28.5 ± 15.3	24.2 ± 14.7	0.12*
sex（Male:Female）	22:21	20:11	0.25**
Morphology of DLM (n) Complete/Incomplete	none	10/21	-
Meniscus Tear (n) (+)/(-)	none	9/22	-
*The Mann–Whitney U test, **Pearson’s χ2 test with Yate’s continuity correction			

The data of various parameters measured by 3D MRI are shown in Table 2. The DLM group (n=31) showed a significantly larger RMT than the non-DLM group (n=43, DLM: 0.26 ± 0.10; non-DLM: 0.10 ± 0.04, p<0.001). Concerning PCM, the DLM group had a significantly larger value than the non-DLM group (DLM: 0.84 ± 0.23; non-DLM: 0.50 ± 0.10, p<0.001). The DLM group had a significantly smaller L/M ratio than the non-DLM group (DLM: 0.66 ± 0.06; non-DLM: 0.74 ± 0.05, p<0.001) (Table 2).

**Table 2:** Comparison of each parameter value between non-DLM and DLM groups. DLM: Discoid lateral meniscus; RMT: Ratio of the meniscus to the tibia; PCM: Percent coverage of the meniscus. The data has been represented as Mean ± SD.

	Non-DLM group (n=43)	DLM group (n=31)	P-value
RMT	0.10 ± 0.04	0.26 ± 0.10	<0.001*
PCM	0.50 ± 0.10	0.84 ± 0.23	<0.001*
L/M ratio	0.74 ± 0.05	0.66 ± 0.06	<0.001*
*The Mann–Whitney U test			

Subgroup analysis between the complete and incomplete DLM groups showed no significant differences in PCM and L/M ratio (Table 3). In addition, subgroup analysis between the cases with and without tears in the DLM group was performed. It was demonstrated that RMT and PCM values were significantly smaller in cases with tears, and the torn DLM showed a significantly larger L/M ratio (Table 4).

**Table 3:** The difference in values of morphological parameters between complete and incomplete DLM groups. DLM: Discoid lateral meniscus; PCM: Percent coverage of the meniscus. The data has been represented as mean ± SD.

	Complete DLM group (n=10)	Incomplete DLM group (n=21)	P-value
PCM	0.97 ± 0.09	0.78 ± 0.24	0.07
L/M ratio	0.65 ± 0.06	0.67 ± 0.07	0.18
Complete discoid: RMT > 0.32			

**Table 4:** The difference in values of morphological parameters associating with the presence of the tear in the DLM group. DLM: Discoid lateral meniscus; RMT: Ratio of the meniscus to the tibia; PCM: Percent coverage of the meniscus. The data has been represented as Mean ± SD.

	DLM tear^+ ^(n=9)	DLM tear^- ^(n=22)	P-value
RMT	0.18 ± 0.12	0.28 ± 0.09	<0.05
PCM	0.65 ± 0.22	0.91 ± 0.18	<0.05
L/M ratio	0.74 ± 0.03	0.63 ± 0.05	<0.001

The inter- and intra-observer ICC values were 0.923 (95％ CI: 0.854-0.951) and 0.911 (95％ CI: 0.848-0.947) for the L/M ratio.

## Discussion

**Figure 3:**
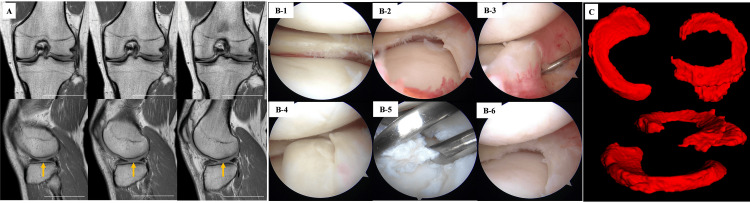
Case presentation. Left lateral meniscus injury in a 23-year-old male; arthroscopic examination and partial meniscectomy were performed. The 2D and 3D views of the meniscus are compared with intraoperative arthroscopic findings. A: Coronal and sagittal knee slices. The characteristic broad image of the DLM is not evident in the coronal slice. Additionally, a continuous meniscus image from front to rear is unconfirmed in the sagittal view. The white bar represents 50 mm. Yellow arrows highlight that the lateral menisci seem to be non-continuous from anterior to posterior. B: Arthroscopic view depicting the discoid lateral meniscus with a flap tear. (1) The flap is not evident in the lateral portal view. (2) The flap is unclear in the view of the medial portal. (3,4) The flap tear is pushed from the lower surface of the anterior horn with a probe and then reduced. (5) Morskite forceps are used for traction and resected using another portal. (6) At first glance, there is no change from (2), but the flap tear was resected. C: The 3D image clearly visualizes the forward-inverted torn meniscus. DLM: Discoid lateral meniscus; 2D: Two dimensional; 3D: Three dimensional.

In this study, we demonstrated the 3D imaging of the DLM with the segmentation of MR images from a common 1.5-T MRI scanner. In addition, to our knowledge, this is the first report revealing the unique feature of DLM, i.e., the L/M ratio in DLM patients is smaller than that in non-DLM patients. DLM has been reported to be especially prevalent in Eastern Asia, including Japan and Korea [2]. Arthroscopy or MRI is considered the standard diagnostic procedure for DLM diagnosis. To date, for diagnosing DLM by MRI, the 2D sequence has been generally used; however, understanding 3D morphology is not easy with 2D MRI, in which information is lost by the slice thickness [13]. The morphology of torn DLM is challenging to interpret due to the limited information obtained from 2D MRI. Meanwhile, 3D sequence and high-resolution MRI have become widespread in recent years [14, 15]. They can reconstruct any cross-section and are promising techniques for evaluating various structures. Ozeki N et al. [16] reported that a major benefit of 3D MRI is its ability to estimate the precise size and shape of the entire meniscus. Therefore, we speculated that the entire image of the meniscus could be captured by imaging it in a 3D sequence and creating a 3D image. Segmentation of cartilage and meniscus often requires manual operation or correction, which requires time and effort [17-19]. However, several software programs have recently been available for reconstructing 3D images with clear interfaces and intuitive segmentation tools [20, 21]. The software we used in this study was user-friendly, had high performance, and was freely available. Thus, it was useful for obtaining a 3D image in a semi-automatic manner. Furthermore, arthroscopy has been widely used as a standard diagnostic procedure for DLM. However, even with arthroscopy, it has been difficult to obtain the entire configuration because of the limited views it offers. Dandy DJ [22] reported that the inverted-type DLM tear looks like a normal LM without a meniscus tear at first glance and that probing is necessary to expose the inverted portion. Our approach has the potential to describe the whole meniscus in each patient, and an inverted-type DLM tear could be detected before the operation (Figure 3). We believe that it is very useful to preoperatively detect not only the inverted posterior horn but also the inverted anterior horn.

3D sizing of the meniscus has been performed in meniscus implantation studies [23], and their data report a median anteroposterior diameter (mean ± SD) of 47.2 ± 3.78 mm for the MM and a median anteroposterior diameter of 35.8 ± 2.95 mm for the LM. Pollard ME et al. [24]. also studied 21 fresh frozen, above-knee amputation specimens with intact cruciate ligaments and menisci and reported the meniscus length. The medial meniscus length was 43.0 ± 3.8 mm, and the lateral meniscus length was 34.0 ± 3.2 mm. The calculated L/M ratios from these data were 75.6% and 79.0%, respectively. These values were very close to the L/M ratio of our non-DLMs. This supports the unique anatomical features of the DLM depicted in the 3D image. Few studies [25] have addressed the anteroposterior diameter of the menisci. Choi NH et al. [25] reported that the insertion center of the posterior horn of the LM is located more medially to the apex of the lateral tibial eminence in DLM than in non-DLM. A limitation of this study is that the insertion center of the posterior horn of the LM was evaluated only on the coronal plane and not on the sagittal or axial planes. The anteroposterior diameter of the DLM must be reduced because the roots' anterior and posterior positions differ from those of the non-DLM. Our study did not evaluate the reason for the lower L/M ratio in the patients with DLM. However, it is speculated that hypoplasia of the lateral compartment of the femur and tibia of DLM patients would lead to a smaller anteroposterior diameter of the tibia and LM.

Although DLM has been described [1, 2, 26] as large and widely covers the articular surface of the tibial plateau, our group reported for the first time that DLM has a smaller L/M ratio than non-DLM. The L/M ratio could potentially be recognized as one of the diagnostic parameters of DLM without being affected by body size. Notably, because the L/M ratio could be affected by tears in the DLM, we need to be careful in evaluating the morphology of the torn DLM. In other words, torn DLM has peripheral rim instability or a natural consequence of spreading due to abnormal tissue properties [27]. These preoperative 3D images could help unveil unique features and design an appropriate partial meniscectomy, although there is no clear answer for the optimal treatment of this entity. Hence, the approach described here is feasible in daily practice without additional cost. Accumulating clinical images and knowledge could accelerate our understanding of this enigmatic disorder and improve clinical outcomes. In the future, we plan to investigate the changes in the 3D morphology of the DLM between preoperative and postoperative conditions using the software after several treatments for this pathology. This study has several limitations. First, the sample size was small. Second, in the DLM group, the presence of a tear significantly increased the L/M ratio; however, including a tear case in the DLM group may be a limitation when comparing the DLM and non-DLM groups. Finally, the significance of the small L/M ratio in the DLM group compared with that in the non-DLM group was not investigated. In addition, the postoperative L/M ratio and clinical results were not investigated. Despite several limitations, the 3D images obtained in this study were useful for describing the unique features of DLM and for interpreting DLM with the aid of previously established quantitative methods. This study also reported that the L/M ratio in patients with DLM was smaller than that in patients without DLM.

## Conclusions

Our approach holds the potential to depict the entirety of the menisci in each patient, and these preoperative 3D images could prove instrumental in revealing unique features and in planning appropriate meniscectomies. The 3D images obtained in this study were useful for describing the unique features of DLM and for interpreting DLM, aided by previously established quantitative methods. Additionally, this study revealed that the L/M ratio in patients with DLM was smaller compared to those without DLM.
